# Impact of Storage Controlled Atmosphere on the Apple Phenolic Acids, Flavonoids, and Anthocyanins and Antioxidant Activity In Vitro

**DOI:** 10.3390/plants11020201

**Published:** 2022-01-13

**Authors:** Aurita Butkeviciute, Jonas Viskelis, Mindaugas Liaudanskas, Pranas Viskelis, Valdimaras Janulis

**Affiliations:** 1 Department of Pharmacognosy, Lithuanian University of Health Sciences, Sukileliu Av. 13, LT-50162 Kaunas, Lithuania; Mindaugas.Liaudanskas@lsmu.lt (M.L.); Valdimaras.Janulis@lsmuni.lt (V.J.); 2 Laboratory of Biochemistry and Technology, Lithuanian Institute of Horticulture, Lithuanian Research Centre for Agriculture and Forestry, Kauno Str. 30, LT-54333 Babtai, Lithuania; Jonas.Viskelis@lammc.lt (J.V.); Pranas.Viskelis@lammc.lt (P.V.)

**Keywords:** *Malus domestica*, polyphenols, atmosphere

## Abstract

Apples are seasonal fruits, and it is important to prepare them adequately for storage and ensure proper storage conditions. In this study, we used ten different apple cultivars: ‘Alva’, ‘Auksis’, ‘Connell Red’, ‘Cortland’, ‘Ligol’, ‘Lodel’, ‘Noris’, ‘Rubin’, ‘Sampion’, and ‘Spartan’. We studied the qualitative and quantitative composition of phenolic compounds in the apple and apple extracts antioxidants activity before placing them in the controlled atmosphere chambers and again at the end of the experiment, eight months later. Different concentrations of O_2_, CO_2_, and N_2_, constant temperature, relative humidity, and removal of endogenous ethylene were continually maintained. HPLC analysis showed that the highest amount of 2265.7 ± 152.5 µg/g of chlorogenic acid was found in apple samples of the ‘Auksis’ cultivar stored under variant IV conditions. Different concentrations of gas in the controlled atmosphere chambers caused changes in antioxidant activity in whole apple and apple peel extracts. In our study, we found that the antioxidant activity of apple extracts varied between samples of different apple cultivars and depended on the composition of the controlled atmosphere. Determining the optimal storage conditions is beneficial to providing the consumers with apples that have a known and minimally altered chemical composition of phenolic compounds and the strongest antioxidant activity, which determine the use of apples in the healthy food chain.

## 1. Introduction

Over the recent years, consumers have been seeking high-nutrition and health-enhancing products, and thus interest in “functional foods” (fruits, berries, etc.) has been growing rapidly [1]. Apples have occupied an important place in the human food chain and to this day remain among the most widely consumed fruits in the world [2]. Around 3000 different apple cultivars are grown worldwide, the fruits of which are used in the food industry to manufacture products, food supplements, and beverages [3].

The nutritional properties of apples are determined by the complex of biologically active compounds and the unique composition of individual secondary metabolites [3]. Phenolic compounds are the predominant group of secondary metabolites in apples [4,5,6], the amount of which in the fruit is determined by the apple cultivar, rootstock, climatic conditions, and agrotechnology [7,8]. Five main groups of phenolic compounds have been identified in apples: hydroxycinnamic acids, flavan-3-ols, flavonols, dihydrochalcones, and anthocyanins [2,3,9]. These groups of biologically active phenolic compounds determine the variety of colour shades and the sensory properties of the apples, which influence the vegetative maturity and quality indicators of the fruit [1,10]. Fruits of different apple cultivars are characterised by their physical and sensory properties, but strong antioxidant activity is also a characteristic property of apple fruits, and it is determined by the complex of phenolic compounds [3,6]. The phenolic compounds in apple fruit act as reducing agents by donating hydrogen, quenching singlet oxygen, acting as chelators, and trapping free radicals, thus protecting the DNA, proteins, lipids, and other macromolecular structures from the damaging effects of free radicals [2]. Phenolic compounds lower blood glucose levels [11,12] and have anticancer [13], anti-inflammatory [14,15], antimicrobial [16,17], antiobesity [18,19], cardioprotective [20,21], and neuroprotective [22] effects. Daily consumption of fruit rich in complexes of biologically active phenolic compounds reduces the risk of chronic and degenerative diseases [6].

Apples are seasonal fruits, thus, after harvesting apples of optimal ripeness [23,24], the fruits are prepared for medium or long-term storage (10–11 months, depending on the apple cultivar) in order to extend the shelf life and to ensure the supply of high-quality and high-nutrition value fruit to the market [25]. To achieve these objectives, apples grown in industrial gardens are increasingly kept in controlled atmospheric conditions [26,27]. Scientific literature describes storage of the fruits in chambers with low-oxygen (about 1 kPa) [28], or ultralow-oxygen (0.5 and 0.7–0.8 kPa) [24], high carbon dioxide (2–3 kPa) [9], and low temperature (0.5–1.0 °C) and high relative humidity (94–96%) conditions [29]. Storage of fruit in an anaerobic environment with low temperature slows down the metabolism [9], ethylene production [30], and fermentation processes [31] in the cells. These factors influence the resistance of the fruit to diseases caused by fungal strains and allow for maintaining the quality of the fruit and prolonging its shelf life [32,33].

With the recent intensive development of horticulture and the production of large quantities of apples, it becomes necessary to store them and extend their shelf life in order to provide the consumers with high-quality and high nutrition value products. Phenolic compounds are an important group of biologically active compounds that determine the nutritional properties of apples and their effect on the prevention of various diseases. In our study, apples were stored under different controlled atmospheric conditions. Studies on the storage conditions of apples in controlled atmosphere chambers are usually limited to assessing changes in the physical and sensory properties of the fruit during storage. The analysis of the scientific literature did not yield any published studies that would provide information on the changes in the qualitative and quantitative composition of individual phenolic compounds in apples and the antioxidant activity of apple sample extracts during storage in controlled atmospheric conditions. The aim of this study was to determine changes in the qualitative and quantitative composition and antioxidant activity of phenolic acids, flavonoids, and anthocyanins in vitro in apples stored in a controlled atmosphere of different compositions. We determined the variation trend in the qualitative and quantitative composition of phenolic acids, flavonoids, and anthocyanins in apple samples during storage in controlled atmospheric conditions and evaluated the ability of apple extracts and phenolic compounds to bind free radicals in vitro. The results obtained during the study provide new knowledge of the optimal storage conditions of apples, which will allow for preserving apples with the least-changed qualitative and quantitative composition of phenolic acids, flavonoids, and anthocyanins, and with minimal reduction of antioxidant activity in vitro.

## 2. Results and Discussion

### 2.1. Variation in the Quantitative Composition of Phenolic Acids in Apple Samples before and after Storage in Controlled Atmospheric Conditions

**Figure 1:**
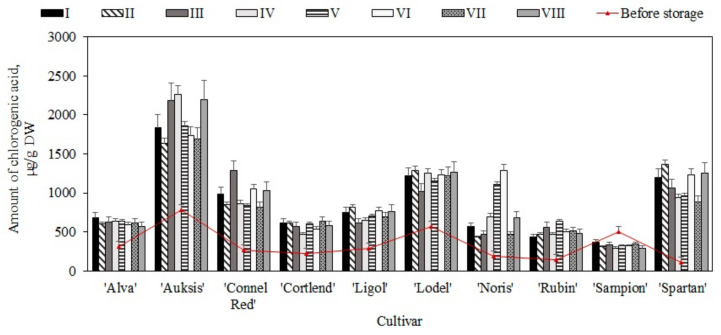
Changes in the amount of chlorogenic acid in whole apple samples before and after storage in controlled atmosphere.

The study showed that before placing the apple samples in the controlled atmosphere chambers (i.e., before the storage), the content of chlorogenic acid in the samples varied from 109.4 ± 10.4 µg/g to 780.4 ± 31.7 µg/g (Figure 1). The statistically significantly highest content of chlorogenic acid (780.4 ± 31.7 µg/g, *p* < 0.05) was found in fruit samples of the ‘Auksis’ apple cultivar, while the lowest content (109.4 ± 10.4 µg/g, *p* > 0.05) was found in fruit samples of the ‘Spartan’ apple cultivar (Figure 1). Chlorogenic acid accounted for between 13.2% and 58.2% of the total amount of phenolic compounds. Tsao et al. found that chlorogenic acid accounted for 21.0–90.0% of the total amount of phenolic compounds in apple samples [34]. Meanwhile, Rana et al. found that the amount of chlorogenic acid in apple samples varied from 106.8 µg/g to 198.9 µg/g [35]. A study by Bars–Cortina et al. showed that chlorogenic acid in red-fleshed and white-fleshed apple samples ranged from 52.3 µg/g to 306.0 µg/g [36]. The results of our study confirm the data obtained by Rana and Bars–Cortina.

The qualitative and quantitative composition of chlorogenic acid was evaluated before placing the apple samples in the storage chambers and again eight months later at the end of the experiment. Chlorogenic acid levels were found to have increased in fruit samples of all (except for ‘Sampion’) apple cultivars stored under controlled atmospheric conditions (variants I–VIII) compared with the levels detected before the storage. Compared to the amount of chlorogenic acid detected before the storage, the content of this compound increased most significantly in the apple samples stored under variant VI conditions. The most significant increase in chlorogenic acid content (from 780.4 ± 35.9 µg/g to 2265.7 ± 152.5 µg/g) was found in apple samples of the ‘Auksis’ cultivar stored under variant IV conditions (Figure 1). The most significant decrease in chlorogenic acid content (from 503.9 ± 31.4 µg/g to 289.9 ± 18.6 µg/g) was found in apple samples of the ‘Sampion’ cultivar stored under variant VIII conditions (Figure 1). Sluis et al. in their study found that after 46 weeks of storage in controlled atmospheric conditions, samples of ‘Jonagold’ apples showed a reduction in chlorogenic acid content, while no significant reduction in chlorogenic acid levels was observed in apple samples of the ‘Golden Delicious’ cultivar [37]. Vondráková et al. found that after three months of storage under ultralow-oxygen (ULO) conditions, the content of phenolic acids in apple samples of the ‘Karneval’ cultivar increased [30]. The researchers found that after seven months of storage under ULO conditions, the amount of phenolic acids decreased significantly but remained close to the amount detected before the storage [30]. In order to prolong the shelf life of apples, researchers often keep the fruit in controlled atmosphere chambers filled with an ethylene inhibitor 1-methylcyclopropene (1-MCP) [38]. MacLean et al. found that in apple samples of the ‘Delicious’ cultivar, the amount of chlorogenic acid decreased during storage in chambers with a controlled atmosphere containing 1-MCP compared with the amount determined during storage without the use of an ethylene inhibitor [39].

Chlorogenic acid is an essential component of biologically active compounds predominant in apple samples and has a strong antioxidant effect [40]. It is thus imperative to determine the dynamics of changes in the quantitative composition of chlorogenic acid during storage under controlled atmospheric conditions and to evaluate the effect of changes in quantitative composition on antioxidant activity in vitro during storage.

### 2.2. Variation in the Quantitative Composition of Flavan-3-ols in Apple Samples before and after Storage in Controlled Atmospheric Conditions

**Figure 2:**
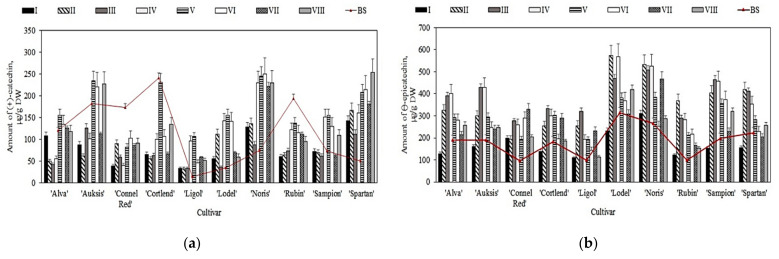
Changes in the amount of monomeric flavan-3-ols in whole apple samples before and after storage in controlled atmosphere: (**a**) Changes in the amount of (+)-catechin; (**b**) Changes in the amount of (−)-epicatechin.

The amounts of monomeric ((+)-catechin and (−)-epicatechin) and oligomeric (procyanidin B1, procyanidin B2, and procyanidin C1) flavan-3-ol compounds detected in samples of apples stored under controlled atmospheric conditions ranged from 25.1% to 68.9% of the total amount of phenolic compounds. Gacnik et al. found that flavan-3-ols in apple samples might account for 46.0–67.0% of the total amount of phenolic compounds [41]. Our study showed that the levels of (+)-catechin and (−)-epicatechin in apple samples before storage in controlled atmosphere chambers varied from 14.5 ± 1.1 µg/g to 240.9 ± 12.4 µg/g and from 95.4 ± 4.2 µg/g to 314.9 ± 23.7 µg/g, respectively (Figure 2). The maximum (+)-catechin content (240.9 ± 12.4 µg/g) was found in apple samples of the ‘Cortland’ cultivar, and the maximum (−)-epicatechin content (314.9 ± 23.7 µg/g) was found in apple samples of the ‘Lodel’ cultivar (Figure 2). Wojdylo et al. found that the content of (+)-catechin in fruit samples of apple cultivars grown in Poland varied from 10.0 µg/g to 720.0 µg/g, and (−)-epicatechin content ranged from 70.0 µg/g to 2760.0 µg/g [42]. The data obtained by these researchers confirm the results of our study.

The quantitative composition of monomeric flavan-3-ols varied in apple samples stored in controlled atmosphere chambers. The total content of individual flavan-3-ols in apple samples stored under controlled atmospheric conditions (variants I–VIII) ranged from 532.6 ± 35.5 µg/g to 1959.7 ± 97.4 µg/g. The content of (+)-catechin was found to have increased in apple samples of ‘Ligol’, ‘Lodel’, ‘Noris’, and ‘Spartan’ cultivars stored under variant I–VIII conditions compared with the amount detected before the storage. The content of (+)-catechin in fruit samples of different apple cultivars stored under variant V conditions increased significantly compared with the amount detected before the storage (Figure 2a). The largest increase in (+)-catechin content (from 14.5 ± 4.1 µg/g to 105.7 ± 12.4 µg/g) was found in apple samples of the ‘Ligol’ cultivar stored under variant V conditions (Figure 2a). The (+)-catechin content was reduced in apple samples of ‘Connell Red’, ‘Cortland’, and ‘Rubin’ cultivars stored under variant I–VIII conditions compared with the amount detected before the storage. The most significant decrease in (+)-catechin content was found in apple samples stored under variant I–III conditions (Figure 2a).

A trend of increasing (−)-epicatechin content was observed during apple storage in controlled atmospheric conditions. Samples of apples stored under variant III conditions showed the most significant increase compared with that before the storage. The largest increase in (−)-epicatechin content (from 99.9 ± 9.5 µg/g to 369.4 ± 26.5 µg/g) was found in apple samples of the ‘Rubin’ cultivar stored under variant II conditions (Figure 2b), while the most significant decrease (from 15.55% to 31.85%) was found in fruit samples of apple cultivars kept under variant I conditions (Figure 2b). Sluis et al. noted that after 46 weeks of storage in controlled atmospheric conditions, apple samples of the ‘Jonagold’ cultivar showed an 18.0% to 40.0% decrease in (+)-catechin content [37]. Stanger et al. found that the content of (+)-catechin and (−)-epicatechin in apple samples of the ‘Galaxy’ cultivar stored for nine months varied from 0.2 µg/g to 2.9 µg/g under dynamic controlled atmospheric conditions and from 0.1 µg/g to 107.9 µg/g under ultralow oxygen conditions [9]. The results of our study confirmed those obtained by Stanger et al.

**Figure 3:**
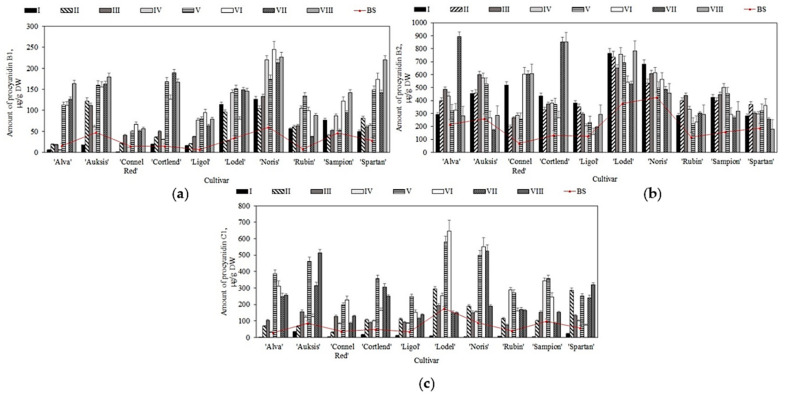
Changes in the amount of oligomeric flavan-3-ols in whole apple samples before and after storage in controlled atmosphere: (**a**) Changes in the amount of procyanidin B1; (**b**) Changes in the amount of procyanidin B2; (**c**) Changes in the amount of procyanidin C1.

The studies had shown that before placing the samples in controlled atmosphere chambers (i.e., before the storage), the quantitative composition of oligomeric flavan-3-ols (procyanidin B1, procyanidin B2, and procyanidin C1) in apple samples ranged from 6.4 ± 1.5 µg/g to 425.7 ± 23.2 µg/g (Figure 3). The highest content of procyanidin B2 (425.7 ± 23.2 µg/g) was detected in apple samples of the ‘Noris’ cultivar, while the lowest content of procyanidin B1 (6.4 ± 1.5 µg/g) was registered in apple samples of the ‘Ligol’ cultivar (Figure 3). Belviso et al. found that the content of procyanidin B2 in samples of apples grown in Italy varied from 18.0 µg/g to 2090.0 µg/g [43]. Piccolo et al. indicated that the amount of procyanidin B1 in apple cultivars grown in Italy varied from 6.0 µg/g to 14.0 µg/g [44]. Meanwhile, Wojdylo et al. found that the content of procyanidin C1 in samples of apples grown in Poland varied from 0.6 µg/g to 970.0 µg/g [42]. The results obtained in these studies confirm the results of our analysis.

In the apple samples stored in the controlled atmosphere chambers, the quantitative composition of procyanidins varied. Compared with the prestorage levels, procyanidin B1 levels increased in apple samples stored under variant V–VIII conditions. The most significant increase in procyanidin B1 content (from 8.1 ± 0.6 µg/g to 134.1 ± 14.2 µg/g) was observed in apple samples stored under variant V conditions (Figure 3a). An increasing trend of procyanidin B2 content was found in apple samples stored under variant I–VIII controlled atmospheric conditions. The most significant increase in procyanidin B2 levels was found under I, VII, and VIII apple storage conditions. In apple samples of the ‘Connell Red’ cultivar, procyanidin B2 levels increased from 69.8 ± 9.8 µg/g to 519.6 ± 22.4 µg/g (under variant I conditions) and from 69.8 ± 9.8 µg/g to 604.8 ± 39.5 µg/g (under variant VI conditions), from 69.8 ± 9.8 µg/g to 603.8 ± 33.2 µg/g (under variant VII conditions), and from 69.8 ± 9.8 µg/g to 607.5 ± 44.6 µg/g (under variant VIII conditions) (Figure 3b). The study showed that procyanidin C1 levels were reduced in apple samples stored under variant I conditions compared with the prestorage levels (Figure 3c). The levels of procyanidin C1 in apple samples stored under variant II–VIII conditions varied. The most significant increase in procyanidin C1 content was found in apple samples stored under variant V conditions, with ‘Alva’ cultivar showing the largest increase from 29.4 ± 5.1 µg/g to 386.4 ± 31.5 µg/g (Figure 3c). Lv et al. in their study found that after four months of cold storage, procyanidin B2 levels in apple peel samples of ‘Amorosa’ and ‘Santana’ cultivars increased from 264.2 µg/g to 277.8 µg/g and from 166.3 µg/g to 203.7 µg/g, respectively [45]. The results of our study confirm their data.

Scientific literature provides data on the multifaceted biological effects of flavan-3-ols. Procyanidin B1 has been shown to have an anti-Alzheimer’s effect [46], (−)-epicatechin offers benefits regarding renal alterations associated with inflammatory or metabolic diseases and increased muscle growth and strength [47,48], and (+)-catechin has antiobesity effects [49]. Catechin, epicatechin, and procyanidin B1 are the main compounds in apples responsible for reducing cholesterol levels [1]. Therefore, it is desirable to determine the optimal storage conditions for apples and quantify the composition of the flavan-3-ol group components to minimise storage-related alterations in the composition of the biologically active compounds in products supplied to the consumers.

### 2.3. Variation in the Quantitative Composition of Flavonols in Apple Samples before and after Storage in Controlled Atmospheric Conditions

**Figure 4:**
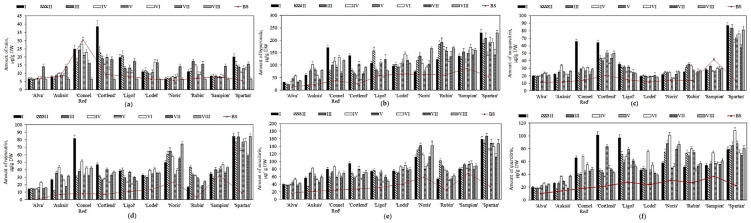
Changes in the amount of flavonols in whole apple samples before and after storage in controlled atmosphere: (**a**) Changes in the amount of rutin; (**b**) Changes in the amount of hyperoside; (**c**) Changes in the amount of isoquercitrin; (**d**) Changes in the amount of reynoutrin; (**e**) Changes in the amount of avicularin; (**f**) Changes in the amount of quercitrin.

Flavonols (rutin, hyperoside, isoquercitrin, reynoutrin, avicularin, and quercitrin) detected in samples of apples stored under controlled atmospheric conditions comprised from 3.8% to 25.3% of the total amount of phenolic compounds. Gacnik et al. found that flavonols could account for 13.0–26.0% of the total phenolic compounds in apple samples [41]. Our study showed that before placing the apple samples in the controlled atmosphere chambers (i.e., before the storage), the amount of the detected individual flavonols in the samples varied from 0.8 ± 0.1 µg/g to 87.1 ± 10.3 µg/g (Figure 4). The highest hyperoside content (87.1 ± 10.3 µg/g) was found in apple samples of the ‘Sampion’ cultivar (Figure 4b), while the lowest amount of reynoutrin (0.8 ± 0.1 µg/g) was detected in the samples of the ‘Alva’ cultivar (Figure 4d). The amounts of flavonols detected in the samples prior to their placement in the controlled atmosphere chambers can be arranged in the descending order as follows: hyperoside > avicularin > quercitrin > isoquercitrin > reynoutrin > rutin.

An increasing trend in flavonol levels was observed in apple samples stored under controlled atmospheric conditions. The total flavonol content in apple samples stored under variant I–VIII controlled atmospheric conditions ranged from 105.8 ± 19.2 µg/g to 658.2 ± 34.3 µg/g. Hyperoside levels in apple samples were found to increase compared with the prestorage levels. The most significant increases in hyperoside were noted in the samples stored under variant I and V conditions. The most significant increase in hyperoside content (from 25.1 ± 5.7 µg/g to 137.3 ± 24.1 µg/g) was found in the ‘Cortland’ cultivar samples stored under variant I conditions (Figure 4b). ‘Sampion’ cultivar samples stored under variant I to VIII conditions showed a decreasing trend in isoquercitrin levels. The content of isoquercitrin in the ‘Sampion’ cultivar samples decreased by 21.1% to 38.7% (Figure 4c). Apple samples of the ‘Alva’ cultivar stored under variant I–VIII conditions showed a significant upward trend in reynoutrin levels. The greatest increases (from 0.8 ± 0.1 µg/g to 15.7 ± 2.6 µg/g and from 0.8 ± 0.1 µg/g to 16.1 ± 3.1 µg/g) were found in the samples of the ‘Alva’ cultivar stored under variant V and VIII conditions, respectively (Figure 4d). The avicularin content in apple samples stored under variant III and IV conditions increased compared to the prestorage levels (Figure 4e). Fruit samples of different apple cultivars stored under variant I–VIII conditions showed an increasing trend in quercitrin content (Figure 4f). The most significant increases in isoquercitrin, avicularin, and quercitrin levels were found in the ‘Spartan’ cultivar samples. Fruit samples of different apple cultivars stored under variant I–VIII conditions showed a decreasing trend in rutin content. The ‘Connell Red’ cultivar samples showed the most significant decreases from 30.6 ± 1.2 µg/g to 16.9 ± 3.5 µg/g (under variant II conditions), from 30.6 ± 1.2 µg/g to 16.3 ± 4.2 µg/g (under variant VII conditions), and from 30.6 ± 1.2 µg/g to 6.4 ± 0.2 µg/g (under variant VIII conditions) (Figure 4a). Lv et al. found that after four months of cold storage, the amounts of hyperoside, isoquercitrin, reynoutrin, avicularin, and quercitrin in apple peel samples of ‘Amorosa’ and ‘Santana’ cultivars increased, while the amount of rutin decreased compared with the amounts detected before the storage [45]. The results of the studies carried out by these researchers confirm the increasing and decreasing trends of flavonol content during the storage of apple samples found in our study.

Flavonol compounds are potent antioxidants with anti-inflammatory, anticancer, anticoagulant, antiallergic, and antiviral effects [44,50]. It is thus important to evaluate the dynamics of the changes in the qualitative and quantitative composition of the flavonol group compounds during the storage of apples and to determine the storage conditions during which the maximum amount of biologically active compounds would be preserved in apples.

### 2.4. Variation in the Quantitative Cmposition of Dihydrochalcone in Apple Samples before and after Storage in Controlled Atmospheric Conditions

**Figure 5:**
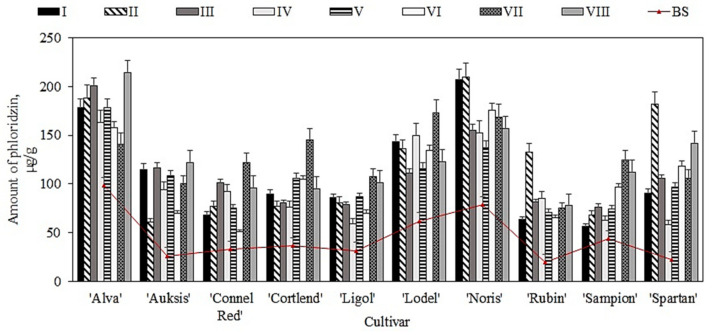
Changes in the amount of phloridzin in whole apple samples before and after storage in controlled atmosphere.

The amount of phloridzin found in the samples of apples stored under controlled atmospheric conditions ranged from 1.6% to 11.6% of the total amount of phenolic compounds. The study showed that before placement of the apple samples in the controlled atmosphere chambers (i.e., before the storage), the amount of phloridzin in the samples varied from 19.5 ± 2.1 µg/g to 78.8 ± 15.2 µg/g (Figure 5). The highest content of phloridzin (78.8 ± 15.2 µg/g) was found in apple samples of the ‘Noris’ cultivar (Figure 5). Piccolo et al. found that the amount of phloridzin in fruit samples of apple cultivars grown in Italy ranged from 10.0 µg/g to 50.0 µg/g [44]. These findings confirm the results of our study.

An increasing trend of phloridzin content was observed in apple samples stored under variant I–VIII conditions. The samples stored under variant II conditions showed the most significant increase in phloridzin content compared with that detected before the storage. The greatest increases in phloridzin content (from 19.5 ± 2.1 µg/g to 132.2 ± 21.2 µg/g and from 22.6 ± 6.1 µg/g to 181.7 ± 32.2 µg/g) were observed in apple samples of ‘Rubin’ and ‘Spartan’ cultivars, respectively, stored under variant II conditions (Figure 5). Lv et al. found that after four months of cold storage, the amount of phloridzin in apple samples of the ‘Amorosa’ cultivar increased from 4.9 µg/g to 18.0 µg/g, while in apple samples of the ‘Santana’ cultivar, it decreased from 60.3 µg/g to 55.2 µg/g [45]. In a study by Stanger et al., the content of phloridzin in apple samples stored for nine months varied from 0.2 µg/g to 22.9 µg/g under dynamic controlled atmosphere conditions and from 0.1 µg/g to 15.6 µg/g under ultralow oxygen conditions [9]. The results of the studies performed by Lv and Stanger confirm the findings of our study.

Qualitative and quantitative analysis of the dihydrochalcone group compounds is essential, as these compounds may be selected as chemotaxonomic markers in the taxonomy of apple species as well as for the identification of apple products and quality assessment [36]. Compounds of the dihydrochalcone group are widespread in the vegetative and generative organs of plants of the *Malus* L. genus while being largely absent in other plant species [3,36]. Phloridzin can inhibit lipid peroxidation and has been proposed as an antihyperglycemic and antihyperlipidemic agent in diabetes and a potential therapeutic agent in obesity [3,36]. It is thus critical to evaluate the storage-related dynamics of the quantitative composition of phloridzin as a biological marker selectively detected in plants of the apple genus and determine the storage conditions during which the amount of phloridzin would change the least.

### 2.5. Variation in the Quantitative Composition of Anthocyanin in Apple Peel Samples before and after Storage in Controlled Atmospheric Conditions

Anthocyanins are a labile group of biologically active compounds whose stability depends on the light intensity, oxygen, temperature, pH, and the action of enzymes [51]. Thus, it is important to evaluate changes in the qualitative and quantitative composition of anthocyanins under controlled atmospheric conditions. The highest amounts of anthocyanins, which determine the variety of colour shades and sensory properties of apples, were found in apple peels. We evaluated peels of fruit samples of different apple cultivars. The peels were separated after the storage of the samples under controlled atmospheric conditions.

The evaluation of the qualitative and quantitative composition of apple peels showed that cyanidin 3-O-galactoside was the predominant anthocyanin in apple peels. Small amounts of cyanidin 3-O-arabinoside chloride, peonidin 3-O-galactoside chloride, and peonidin 3-O-arabinoside chloride were found in apple peel samples of the studied cultivars. Wang et al. found that cyanidin 3-O-galactoside might account for 80.0–94.0% of the total amount of anthocyanins [5]. There is evidence in the scientific literature that the red colour of the apples is determined by anthocyanins, which may account for 1.0 to 3.0% of the total amount of phenolic compounds in red apple peel samples [52].

**Figure 6:**
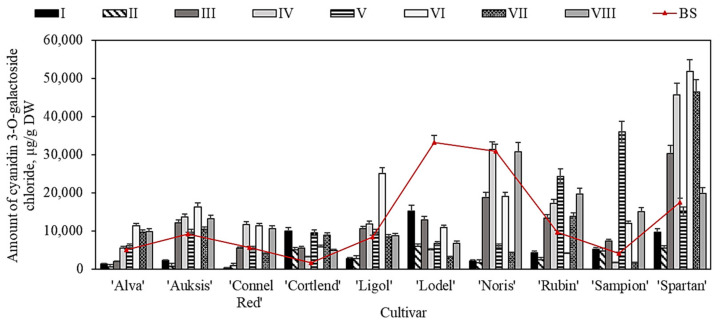
Changes in the cyanidin 3-O-galactoside chloride in the apple peel samples before and after storage in controlled atmosphere.

Our study showed that the amount of cyanidin 3-O-galactoside chloride in apple peel samples ranged from 1678.9 ± 85.1 µg/g to 33,183.6 ± 941.5 µg/g before the samples were placed in the controlled atmosphere chambers (i.e., before the storage) (Figure 6). The highest content of cyanidin 3-O-galactoside chloride (33,183.6 ± 941.5 µg/g) was found in apple peel samples of the ‘Lodel’ cultivar (Figure 6). Liu et al. found that the amount of cyanidin 3-O-galactoside chloride in apple peel samples may vary up to 19,590.0 µg/g in the ‘Golden delicious’ cultivar and up to 71,410.0 µg/g in the ‘Pink Lady’ cultivar [10]. These researchers also found that red apple samples contained 3-fold higher levels of cyanidin 3-O-galactoside than the apple samples of other colours [10].

The quantitative composition of cyanidin 3-O-galactoside chloride varied in apple peel samples stored in controlled atmosphere chambers. Apple peel samples of different cultivars (except for ‘Cortland’ and ‘Sampion’) kept under variant I and II conditions showed the most significant decrease in the amount of cyanidin 3-O-galactoside chloride. The levels dropped by 45.0% to 94.8% (variant I conditions) and by 67.1% to 94.5% (variant II conditions) (Figure 6). The most significant increase in cyanidin 3-O-galactoside chloride was observed in apple peel samples of different cultivars stored under variant V–VI conditions. The largest (from 1678.9 ± 85.1 µg/g to 9424.2 ± 456.9 µg/g, and from 4138.5 ± 225.4 µg/g to 35,891.9 ± 1001.5 µg/g) was observed in apple peel samples of ‘Cortland’ and ‘Sampion’ cultivars, respectively, stored under variant V conditions (Figure 6). During storage, the quantitative and qualitative composition of anthocyanins in fruits, vegetables, and berries is determined by the atmosphere’s composition in the storage chambers. Studies have shown that normal atmospheric conditions (21% of O_2_ and 0.03% of CO_2_) can influence the qualitative and quantitative composition of anthocyanins [53]. This finding has been confirmed by Krupa and Tomala [54], Khorshidi et al. [55], and Zhang et al. [56]. They found that storing blackberries, sweet cherries, cherries, and strawberries under normal atmospheric conditions resulted in a decreasing trend of anthocyanin content compared with the amount of anthocyanins in fruits stored under modified atmospheric conditions [57].

Recently, anthocyanin compounds have been of interest for two main reasons: first, the introduction of anthocyanins as high value-added natural pigments in the food and beverage industry, and second, the presence of fruit or other products enriched with anthocyanins, which have multifaceted biological effects [5]. Anthocyanins have been found to have strong antioxidant properties [5,52]. Tsao et al. found that cyanidin 3-O-galactoside has the most potent antioxidant activity of all anthocyanins detected in apple samples [58]. In medical practice, anthocyanins can be used to prevent diabetes, cancer, bacterial infections, visual disturbances, and cardiovascular diseases [51]. It is thus of great importance to identify trends in the variation in the qualitative and quantitative composition of anthocyanins in apple samples during storage and to provide the consumers with high nutritional value products.

### 2.6. Hierarchical Cluster Analysis of Phenolic Compounds

**Figure 7:**
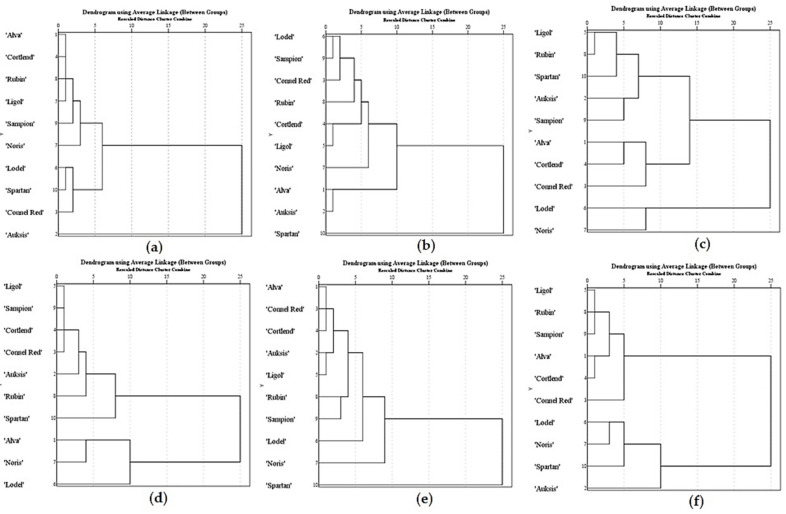
Hierarchical cluster analysis of the identified phenolic compounds in apple samples stored under controlled atmosphere: (**a**) Dendrogram of phenolic acid; (**b**) Dendrogram of flavonols; (**c**) Dendrogram of flavan-3-ols; (**d**) Dendrogram of dihydrochalcone; (**e**) Dendrogram of anthocyanins; (**f**) Dendrogram of the total amount of phenolic compounds.

Determining the optimal storage chamber conditions for apple cultivars is crucial for finding and recommending those that would result in the least change in the chemical composition of biologically active compounds in the fruit samples during storage and would, therefore, preserve the quality and extended shelf life of the fruit. We systematized the results of the analysis of the variation in the qualitative and quantitative composition of phenolic compounds in samples of different apple cultivars stored under controlled atmospheric conditions. We conducted a hierarchical cluster analysis of phenolic compounds detected in samples of different apple cultivars stored under controlled atmospheric conditions. The results of the analysis are presented in Figure 7.

The amounts of chlorogenic acid in apple samples stored under different controlled atmospheric conditions were divided into three clusters. Apple samples assigned to cluster I (1, 4, 5, and 7–9) stored under the conditions of variants I–VIII contained the lowest amounts of chlorogenic acid. Average amounts were detected in apple samples assigned to cluster II (3, 6, and 10). Cluster III included the highest levels of chlorogenic acid detected in apple samples of the ‘Auksis’ cultivar stored under different controlled atmospheric conditions (Figure 7a). The total amounts of flavonols in apple samples stored under different controlled atmospheric conditions were divided into three clusters. Average amounts of flavonols were detected in apple samples assigned to cluster I (3–9). The smallest amounts of flavonols were detected in apple samples of ‘Alva’ and ‘Auksis’ cultivars assigned to cluster II. The largest amounts were found in apple samples of the ‘Spartan’ cultivar assigned to cluster III (Figure 7b). The total amounts of flavan-3-ols in apple samples stored under different controlled atmospheric conditions were also divided into three clusters. Average amounts of flavan-3-ols were detected in apple samples assigned to cluster I (2, 5, and 8–10). The lowest amounts were detected in apple samples assigned to cluster II (1, 3, and 4), and the highest were found in apple samples of ‘Lodel’ and ‘Noris’ cultivars assigned to cluster III (Figure 7c). The amounts of phloridzin in apple samples stored under controlled atmospheric conditions were divided into two clusters. Average and below-average levels of phloridzin were found in apple samples assigned to cluster I (2–5 and 8–10). The highest amounts of phloridzin were found in apple samples of ‘Alva’, ‘Lodel’, and ‘Noris’ cultivars assigned to cluster II (Figure 7d). The content of cyanidin 3-O-galactoside in apple samples stored under different controlled atmospheric conditions was also divided into two clusters. Average and below-average levels of cyanidin 3-O-galactoside were found in apple samples of cluster I (1–9). The highest levels of cyanidin 3-O-galactoside were found in apple samples of the ‘Spartan’ cultivar assigned to cluster II (Figure 7e). The total amounts of individual phenolic compounds in apple samples stored under different controlled atmospheric conditions were divided into two clusters. Average and below-average total amounts of individual phenolic compounds were detected in apple samples assigned to cluster I (1, 3–5, 8, and 9). The highest total amounts of individual phenolic compounds were detected in apple samples of ‘Auksis’, ‘Lodel’, ‘Noris’, and ‘Spartan’ cultivars assigned to cluster II (Figure 7f).

Changes in the qualitative and quantitative composition of phenolic compounds in apples during storage under controlled atmospheric conditions are caused by a complex of external and internal factors such as the apple cultivar, geographic location of the orchards, irrigation systems, seasonal variability, tree age, harvest time, treatment with 1-methylcyclopropene, concentrations of CO_2_ and O_2_, storage temperature, duration of storage, and the timing of CA application [33,38]. The gas composition in the chambers is an essential factor in determining changes in the quality and nutritional value of fruits and vegetables during storage. In cells of fruit kept under low oxygen conditions, fruit metabolism [9], respiration [59], ethylene production [30], and fermentation processes [31] slow down. Maintaining low oxygen concentrations in controlled atmosphere chambers increases fruit resistance to diseases caused by fungal strains [25]. The optimal composition of the controlled atmosphere gases allows for preserving the quality of the fruit and prolonging their shelf life [32,33].

Horvitz et al. found that the synthesis of phenolic compounds continued after fruits and vegetables were harvested [60]. Changes in fruit storage conditions after harvesting cause abiotic stress, which results in the fruit starting to produce and accumulate secondary metabolites [61,62]. Research has shown that changes in the amount of phenolic compounds in fruits and vegetables depend on atmospheric composition (O_2_ and CO_2_ concentrations) and temperature [63,64,65]. High concentrations of CO_2_ during storage can activate enzymes that cause the degradation of phenolic compounds [63,66]. Studies have shown that low O_2_ and high CO_2_ concentrations during storage may be an abiotic stressor, simultaneously influencing the synthesis of phenolic compounds and increasing their quantitative composition [53,67]. Haffner et al. reported that the content of anthocyanins in red raspberry samples stored under controlled atmospheric conditions was unchanged compared to that detected in fresh berries [68]. Veazie and Collins found an increase in anthocyanin levels in samples of blackberries stored under controlled atmospheric conditions at 2 °C [69]. Romero et al. found that anthocyanin levels increased in grape samples stored under controlled atmospheric conditions at high CO_2_ concentrations and low (0 °C) temperatures [70].

The analysis of apple samples stored for eight months under different controlled atmospheric conditions provided valuable scientific knowledge on the variation in the qualitative and quantitative composition of phenolic acids, flavan-3-ols, flavonols, dihydrochalcones, and anthocyanins. Determining the optimal storage conditions is needed to provide consumers with apples that have a known chemical composition of phenolic compounds, which determines their nutritional value, quality, and use in the healthy food chain, as well as the development of innovative foods.

### 2.7. Variation of In Vitro Antioxidant Activity of Apple Extracts before and after Storage in Controlled Atmospheric Conditions

One of the main mechanisms of antioxidant activity in vitro and in vivo found in phenolic compounds detected in fruit and vegetable samples is the ability to scavenge free radicals [3,71]. The antioxidant activity of phenolic compounds is determined by hydroxyl groups and their redox properties, which allow them to act as reducing agents, hydrogen ion donors, singlet oxygen quenchers, or metal ion chelators [72]. Oxidative stress is associated with various chronic and neurodegenerative diseases [71]. For this reason, the diet should include fruits and vegetables that undergo minimal changes in antioxidant activity during storage. One of the objectives of our study was to evaluate the effect of storage under different controlled atmospheric conditions on the ability of whole apple ethanol extracts and apple peel hydrochloric acid solution in ethanol extracts to scavenge free DPPH radicals in vitro.

**Figure 8:**
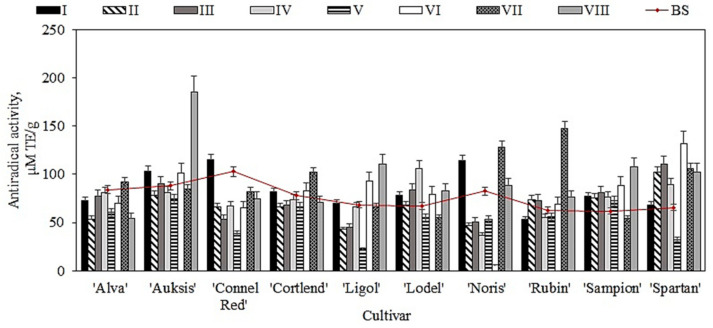
Antioxidant activity of whole apple samples before and after storage in controlled atmosphere evaluated via the DPPH free radical scavenging technique.

Our study showed that before the storage, the antiradical activity of whole apple extracts in different cultivars varied from 61.9 ± 2.4 μM TE/g to 103.1 ± 12.3 μM TE/g (Figure 8). The whole apple extract of the ‘Connell Red’ cultivar was found to have the strongest DPPH free radical scavenging properties (103.1 ± 12.3 μM TE/g) (Figure 8). Faramarzi et al., using the DPPH technique, found that the antiradical activity of 67 apple samples of different cultivars ranged from 10.1 μM TE/g to 129.1 μM TE/g [71]. The data obtained by their research confirm the results of our study.

Our analysis showed that different concentrations of oxygen, carbon dioxide, and nitrogen gas in controlled atmosphere chambers caused changes in the antioxidant activity of whole apple ethanol extracts. An increasing tendency of antioxidant activity was found in whole apple extracts of different cultivars kept under the conditions of variants I, VII, and VIII. The most significant increases in antioxidant activity were found in apple samples of ‘Rubin’ and ‘Auksis’ apple cultivars—from 62.9 ± 3.4 μM TE/g to 147.4 ± 11.5 μM TE/g (under variant VII conditions) and from 88.2 ± 3.5 μM TE/g to 185.3 ± 11.6 μM TE/g (under variant VIII conditions) (Figure 8). Meanwhile, significant reductions in antioxidant activity were observed in apple extracts (except for the ‘Sampion’ cultivar) kept under variant V conditions. The antioxidant activity of apple extracts stored under these conditions decreased by 10.7% to 67.2%, compared with the activity found before the storage. The antioxidant activity of apple extracts of the ‘Noris’ cultivar decreased by 92.9% compared with the activity before the storage (Figure 8). Ma et al. found that after six months of storage, the ability of apple peel extracts to scavenge DPPH radicals decreased by 9.0% to 27.0% [73].

**Figure 9:**
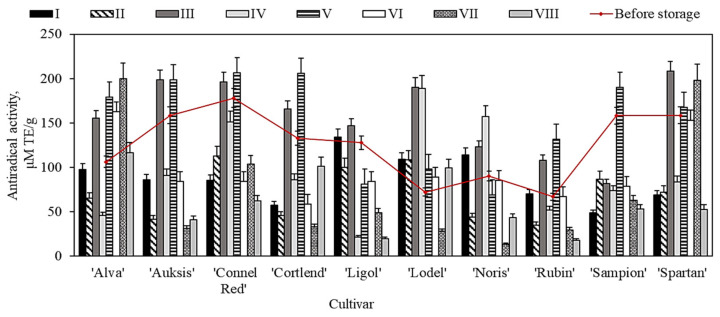
Antioxidant activity of apple peel samples before and after storage in controlled atmosphere evaluated via the DPPH free radical scavenging technique.

Our study showed that before the storage, the antiradical activity of apple peel extracts in different cultivars varied from 66.9 ± 1.4 μM TE/g to 178.1 ± 22.4 μM TE/g (Figure 9). The apple peel extract of the ‘Connell Red’ cultivar had the strongest DPPH free radical scavenging activity (178.1 ± 22.4 μM TE/g) (Figure 9). Asataf et al. found that the DPPH free radical scavenging activity of pomegranate fruit extract varied from 115.7 μM TE/g to 850.1 μM TE/g [12]. Ma et al. found that apple peel extracts had a stronger antiradical activity than apple flesh extracts [73]. The stronger antioxidant activity of apple peels is probably due to the higher content of phenolic compounds in the peel than in flesh samples [73].

The antioxidant activity of apples stored under different atmospheric conditions varied. An increasing tendency of antioxidant activity was found in apple peel extracts stored under variant III and V conditions. The most significant increase was found in apple peel extracts of the ‘Ligol’ cultivar, from 72.2 ± 7.3 μM TE/g to 190.3 ± 16.8 μM TE/g (variant III conditions) and from 72.2 ± 7.3 μM TE/g to 188.6 ± 21.4 μM TE/g (variant IV conditions) (Figure 9). Apple peel samples stored under variant II, VII, and VIII conditions showed a significant reduction in antioxidant activity compared with that observed before the storage. Samples of the ‘Noris’ cultivar showed the most significant (by 84.7%) decrease in antioxidant activity compared with the activity found before the storage (Figure 9). Sudhereen et al., using the DPPH technique, found that red apple extracts showed twice as strong antiradical activity as green apple extracts [74].

### 2.8. Principal Component Analysis

**Figure 10:**
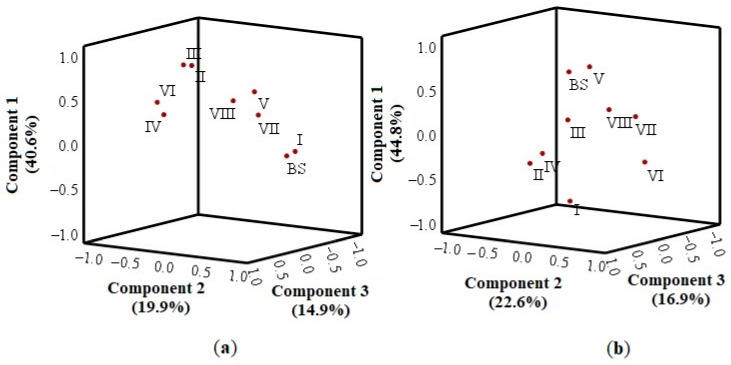
Analysis of the principal components of the antioxidant activity: (**a**) The principal components of the antioxidant activity of the whole apple stored under controlled atmospheric conditions; (**b**) The principal components of the antioxidant activity of apple peel stored under controlled atmospheric conditions.

We analyzed the principal components of the antioxidant activity determined by applying the DPPH free radical scavenging technique in different whole apple and apple peel extracts stored under controlled atmospheric conditions. The results of the antioxidant activity evaluation of whole apple extracts stored under controlled atmospheric conditions were divided into three main components, which explain 75.4% of the total dispersion of the studied data (Figure 10a). The results of the antioxidant activity evaluation of apple peel extracts were also divided into three main components, which explain 84.3% of the total dispersion of the studied data (Figure 10b).

The results of the principal component analysis of the antioxidant activity of whole apple extracts stored under controlled atmospheric conditions determined by the DPPH free radical scavenging technique are shown in Figure 10a. The analysis showed that there was a very strong positive correlation of the antioxidant activity of whole apple extracts stored under variant II (0.832) and variant III (0.852) conditions and a moderately strong correlation of the antioxidant activity of whole apple extracts stored under variant V (0.550) and variant VIII (0.527) conditions with the first component describing 40.6% of the total data dispersion (Figure 10a). The antioxidant activity of whole apple extracts before the storage (0.831) and the antioxidant activity of the whole apple extracts stored under variant I (0.946) conditions very strongly positively correlated with the second component describing 19.9% of the data dispersion (Figure 10a). The antioxidant activity of the whole apple extracts stored under variant VII (0.914) and variant IV (0.779) conditions strongly positively correlated with the third component describing 14.9% of the dispersion. In contrast, the correlation of the antioxidant activity of whole apple extracts stored under variant VI (0.640) conditions was moderately strong (Figure 10a).

The results of the principal component analysis of the antioxidant activity of apple peel extracts stored under controlled atmospheric conditions determined by the DPPH free radical scavenging technique are shown in Figure 10b. The analysis showed that the antioxidant activity of apple peel extracts before the storage (0.719) and those stored under variant V conditions (0.949) very strongly positively correlated, and the antioxidant activity of apple peel extracts stored under variant I conditions very strongly negatively (−0.861) correlated with the first component describing 44.8% of the total data dispersion (Figure 10b). The antioxidant activity of apple peel extracts stored under variant VI (0.958) and variant VII (0.935) conditions very strongly positively correlated with the second component describing 22.6% of the data dispersion (Figure 10b). The antioxidant activity of apple peel extracts stored under variant II (0.802), variant IV (0.710), and variant VIII (0.825) conditions very strongly positively correlated with the third component describing 16.9% of the dispersion. In contrast, the correlation of the antioxidant activity of apple peel extracts stored under variant III conditions was moderately strong (0.610) (Figure 10b).

The antioxidant capacity of fruit and vegetable samples depends on environmental conditions, temperature, UV radiation, exposure to bacterial and fungal strains, and gas composition during storage [25,75,76]. Phenolic compounds are important botanical secondary metabolites with biological effects and the ability to scavenge free radicals. Phenolic compounds of different groups have been found to have different antioxidant properties. Feng et al. studied apple samples and found that chlorogenic acid has stronger antioxidant activity than quercetin, gallic acid, or α-tocopherol, but its antioxidant activity is weaker than that of quercetin 3-O-rutinoside (rutin) [77]. Previous studies showed that catechin and quercetin are the main flavonoids that contribute to the antioxidant potential of apples [6]. Apple peel contains higher levels of phenolic compounds compared with those found in apple flesh samples [76,78]. Flavonols and anthocyanins are usually found in the peel, while flavanols, dihydrochalcones, and hydroxycinnamic acids are the major polyphenol groups found in apple flesh [48]. Li et al. provided evidence that apple peel is directly exposed to (a) biotic stress, which results in a faster synthesis of phenolic compounds and a stronger antioxidant activity of apple peel extracts compared with that observed in apple flesh samples [79].

Researchers have found that during long-time cold storage, DPPH radical scavenging activity in apple peels was more stable than that in apple flesh [73]. The decrease in the antioxidant activity of apples during storage may result from an attack by reactive oxygen species or polymerization of the monomeric phenolic compounds [80]. A decrease in apples’ antioxidant activity (DPPH radical scavenging activity) after storage for six months was also reported by Kolniak–Ostek et al. [81]. The decrease in DPPH radical scavenging activity may be attributed to a decrease in the concentrations of phenolics and flavonoids during storage since antioxidant activity closely correlates with the presence of phenolics and flavonoids [73]. The increase in antioxidant activity was probably due to the synthesis of phenolic compounds, which may be related to induced stress metabolism during cold storage and in a regular or O_2_-enriched atmosphere. These results were consistent with those reported by Zheng et al., who observed significantly higher total phenolic content and DPPH radical scavenging activity in high O_2_-treated Chinese bayberry from day six to the end of storage [82]. However, Fawbush et al. showed that antioxidant activity in apple samples of the ‘Empire’ cultivar remained stable during storage for four-and-a-half months in an ambient atmosphere and during nine months of storage in a controlled atmosphere [83]. Awad and de Jager also found that flavonoids in apples were relatively stable during storage under ultralow oxygen or regular storage conditions and even during shelf life [84].

In our study, we found changes in the antioxidant activity of whole apple and apple peel extracts of samples of different cultivars, as well as changes influenced by the composition of the gases in a controlled atmosphere. The results of our study suggest that selecting the optimal conditions of the controlled atmosphere allows for storing and providing the consumers with apples with minimal changes in the qualitative and quantitative composition of biologically active compounds and the strongest antioxidant activity.

## 3. Materials and Methods

### 3.1. Plant Materials

In this study, we used ten different apple cultivars: ‘Alva’, ‘Auksis’, ‘Connell Red’, ‘Cortland’, ‘Ligol’, ‘Lodel’, ‘Noris’, ‘Rubin’, ‘Sampion’, and ‘Spartan’. Apple samples were prepared at the Institute of Horticulture (Babtai, Lithuania), a branch of the Lithuanian Research Center for Agriculture and Forestry (coordinates: 55°60′ N, 23°48′ E). The study was conducted during 2019–2020.

### 3.2. Chemicals and Solvents

All solvents, reagents, and standards used were of analytical grade. Of these, 99.9% acetonitrile, 36.5–38% hydrochloric acid, >98% formic acid, cyanidin 3-O-galactoside chloride, cyanidin 3-O-arabinoside chloride, peonidin 3-O-galactoside chloride, and peonidin 3-O-arabinoside chloride were obtained from Sigma-Aldrich GmbH (Bethesda, MD, USA). Hyperoside, rutin, quercitrin, phloridzin, procyanidin B1, procyanidin B2, and chlorogenic acid standards were purchased from Extrasynthese (Genay, France), reynoutrin, (+)-catechin, and (−)-epicatechin were obtained from Sigma-Aldrich GmbH (Steinheim, Germany), and avicularin, procyanidin C1, and isoquercitrin were purchased from Chromadex (Santa Ana, CA, USA). Additionally, 96% ethanol was obtained from Stumbras AB (Kaunas, Lithuania), and 2,2-diphenyl-1-picrylhydrazyl (DPPH) was purchased from Vaseline-Fabrik Rhenania (Bonn, Germany). Purified deionized water used in the tests was prepared with the Milli-Q^®^ (Millipore, Bedford, MA, USA) water purification system.

### 3.3. Controlled Atmospheric Conditions during Apple Storage

Controlled atmospheric conditions (Table 1) during apple storage were prepared as described by Butkeviciute et al. [85].

### 3.4. Preparation of Apple and Apple Peel Extracts

Samples of whole apples and apple peels were prepared as described by Butkeviciute et al. [86]. Extracts of whole apple samples were prepared as described by Butkeviciute et al. [87]. Extracts of apple peel samples were produced: 2.5 g of the lyophilized powder (exact weight) was weighed, added to 25 mL of 2% hydrochloric acid solution in 70% (*v*/*v*) ethanol, and was extracted in an ultrasonic bath Sonorex Digital 10 P (Bandelin Electronic GmbH & Co. KG, Berlin, Germany) at room temperature for 20 min. The extraction conditions were chosen based on the results of the tests for setting the extraction conditions. The obtained extract was filtered through a paper filter, and the residue on the filter was washed in a 25 mL flask until the exact volume was reached.

### 3.5. Evaluation of Phenolic Acid and Flavonoids by HPLC-PDA

Qualitative and quantitative high-performance liquid chromatography (HPLC) analyses of phenolic acid and flavonoids in whole apple extracts were performed by applying the method described by Liaudanskas et al. [88].

### 3.6. Evaluation of Anthocyanins by HPLC-PDA

Evaluation of anthocyanins was performed according to the methodology for analysing anthocyanins in dry blueberry extract provided in the European Pharmacopoeia (Ph. Eur. 04/2019:2394). A chromatograph equipped with a Waters 2998 (Waters, Milford, MA, USA) PDA detector was used for the HPLC analysis. Chromatographic separations were carried out using an ACE (5 μm, C18, 250 × 4.6 mm^2^ inner diameter) column. The column was operated at a constant temperature of 30 °C. The volume of the analyzed extract was 10 μL. The flow rate was 1 mL/min. The mobile phase consisted of anhydrous formic acid solution in water (8.5:91.5 *v*/*v*) (solvent A) and a mixture of anhydrous formic acid, acetonitrile, methanol, and water (8.5:22.5:22.5:41.5 *v*/*v*/*v*/*v*) (solvent B). Gradient variation: 0–35 min 93–75% A, 35–45 min 75–35% A, 45–46 min 35–0% A, 46–50 min 0% A, and 0–35 min 7–25% B, 35–45 min 25–65% B, 45–46 min 65–100% B, and 46–50 min 100% B. The identified anthocyanins were quantified according to calibration curves of the standards (cyanidin 3-O-galactoside chloride, cyanidin 3-O-arabinoside chloride, peonidin 3-O-galactoside chloride, and peonidin 3-O-arabinoside chloride). All the identified anthocyanins were quantified at λ = 535 nm wavelength.

### 3.7. Antioxidant Activity Assays

Antioxidant activities of the whole apple and apple peel extracts were evaluated via spectrophotometric assays of DPPH free radical scavenging in vitro using a spectrophotometer (Spectronic CamSpec M550, Garforth, UK). The DPPH free radical scavenging assay was the following: 3 mL of DPPH solution were mixed with 10 μL of whole apple and apple peel extracts. A decrease in absorbance was measured at λ = 517 nm [89]. The calculation of the antioxidant activity of the extract of whole apple and apple peel samples was the following: the antioxidant activity of the extracts was calculated from the Trolox calibration curve and expressed as a μM Trolox equivalent (TE) per gram of the absolutely dry weight (DW). TE was calculated according to the formula TE = c × *V*/m (μM/g), where c was the concentration of Trolox established from the calibration curve (in μM), *V* was the volume of the apple extract (in L), and m was the weight (precise) of the lyophilized apple powder (in g).

### 3.8. Statistical Analysis

The analysis of the HPLC data was performed using Microsoft Office Excel 121 (Microsoft, Redmond, WA, USA) and SPSS, version 25.0 (SPSS Inc., Chicago, IL, USA) software. All the results obtained during the HPLC analysis were presented as means of three consecutive test results and standard deviations. Univariate analysis of variance (ANOVA) was applied to determine whether the differences between the compared data were statistically significant. The hypothesis about the equality of variances was verified by applying Levine’s test. If the variances of independent variables were found to be equal, Tukey’s multiple comparison test was used. The differences were regarded as statistically significant at *p* < 0.05. The comparison of the chemical composition between the apple samples was carried out by applying the hierarchical cluster analysis, using the squared Euclidean distance. Principal component analysis was performed as well. Pearson’s correlation coefficients: 0 < |r| ≤ 0.3 was regarded as a weak correlation, 0.3 < |r| ≤ 0.7 was a moderate correlation, and 0.7 < |r| ≤ 1 was a strong correlation [90].

## 4. Conclusions

The study presents the qualitative and quantitative composition of phenolic compounds in apples stored in variants I–VIII of controlled atmospheric conditions. The study showed that chlorogenic acid levels increased in samples of all (except for ‘Sampion’) apple cultivars stored under controlled atmospheric conditions (variants I–VIII) compared with the levels determined before the storage. In the apple samples stored in controlled atmosphere chambers, the quantitative composition of flavan-3-ols varied. An increasing trend in the amount of individual flavonols and phloridzin was observed in apple samples stored under controlled atmospheric conditions. The most significant increase in cyanidin 3-O-galactoside chloride was found in samples of apple peel of different cultivars stored under variant V and VI conditions. Different concentrations of O_2_, CO_2_, and N_2_ gas in the controlled atmosphere chambers caused changes in antioxidant activity in whole apple and apple peel extracts. The analysis revealed an increasing trend of antioxidant activity in whole apple and apple peel extracts.

Studies of apple samples stored for eight months under different controlled atmospheric conditions provided valuable scientific knowledge on the variation in the qualitative and quantitative composition of phenolic acids, flavan-3-ols, flavonols, dihydrochalcones, and anthocyanins. In our study, we found that the antioxidant activity of apple extracts varied between samples of different apple cultivars and depended on the composition of the controlled atmosphere. Determining the optimal storage conditions is essential to provide the consumers with apples that have a known and minimally altered chemical composition of phenolic compounds and the strongest antioxidant activity, which determine the use of apples in the healthy food chain and the development of innovative products.

## Figures and Tables

**Table 1:** Composition of controlled atmosphere in chambers.

Variant	Amount of Oxygen (O_2_), %	Amount of Carbon Dioxide (CO_2_), %	Amount of Nitrogen (N_2_), %	Relative Humidity, %	Temperature, °C
I	21	0.03	78.97	95 ± 3	+1.5 ± 0.5
II	5	1	94		
III	5	3	92		
IV	5	5	90		
V	5	7	88		
VI	1	3	96		
VII	10	3	87		
VIII	20	3	77		

## Data Availability

All datasets generated for this study are included in the article.
